# A new receptor tyrosine kinase inhibitor, icotinib, for patients with lung adenocarcinoma cancer without indication for chemotherapy

**DOI:** 10.3892/ol.2014.2386

**Published:** 2014-07-28

**Authors:** XIAO ZHENG, GUAN LIU, SHENGYE WANG, YUNLI ZHANG, WENLONG BAO, DEHOU DENG, WEIMING MAO, MEIYU FANG

**Affiliations:** 1 Department of Radiation Oncology, Zhejiang Cancer Hospital, Hangzhou, Zhejiang 310021, P.R. China; 2 Department of Surgical Oncology, Zhejiang Cancer Hospital, Hangzhou, Zhejiang 310021, P.R. China; 3 Integrated Chinese Traditional Medicine and Western Medicine, Zhejiang Cancer Hospital, Hangzhou, Zhejiang 310021, P.R. China; 4 Zhejiang Thoracic Oncology Institute, Zhejiang Cancer Hospital, Hangzhou, Zhejiang 310021, P.R. China

**Keywords:** non-small cell lung cancer, adenocarcinoma, icotinib hydrochloride, performance status

## Abstract

Epidermal growth factor receptor (EGFR) is an important therapeutic target in lung cancer. Gefitinib and erlotinib, two reversible EGFR receptor tyrosine kinases inhibitors (TKIs), have been approved for the treatment of patients with metastatic non small-cell lung cancer. Icotinib, which is a selective EGFR-TKI, provides a similar efficacy to gefitinib. The present study aimed to investigate the survival and safety of icotinib in patients with lung adenocarcinoma with a poor performance status (PS). A total of 42 cases of lung adenocarcinoma, including 35 females and 7 males, were enrolled. Icotinib was used as the first-line of treatment due to poor PS of the patient or a more advanced age. Icotinib (125 mg) was orally administered three times per day. The overall response rate and disease control rates were 33.3 and 85.7%, respectively. The median survival time was 13.0 months (95% CI, 5.6–20.4), The median progression-free survival time was 7.0 months, and the 1-year survival rate was 71.4%. A total of 79% of patients had an improved PS following icotinib treatment. Grade 1 to 2 rashes and diarrhea were the most frequent side effects. One patient succumbed during the study due to interstitial pneumonia. In conclusion, this is the first study indicating that patients with lung adenocarcinoma and poor PS may benefit from first-line icotinib therapy, but should be cautious of the occurrence of interstitial lung disease.

## Introduction

Lung cancer has the highest mortality rate of all cancers worldwide (1). A total of 70–75% of all lung cancers are non small-cell lung cancer (NSCLC) with two-thirds presenting with locally advanced or metastatic disease at diagnosis. Treatment for these patients includes chemotherapy, radiotherapy and best supportive care (BSC) (2). Numerous efforts have been made to improve the treatment efficacy for advanced NSCLC.

Receptor tyrosine kinases, a family of transmembrane proteins, are important factors in cell signal transduction. These kinases control growth factor signal transmission from the cell surface to intracellular processes, and administrate critical cellular activities such as growth, differentiation, angiogenesis and inhibition of apoptosis. These signaling pathways promote the proliferation and formation of metastases of malignant cells. The epidermal growth factor receptor (EGFR) tyrosine kinase family is part of this family of receptor tyrosine kinases (3). Gefitinib and erlotinib are small-molecule tyrosine kinase inhibitors (TKIs) that target EGFR, and such inhibitors were the first targeted drugs to enter clinical use for the treatment of lung cancer (4,5). These two drugs are the standard first-line treatment for patients with advanced NSCLC whose tumors have activating EGFR mutations. This treatment option has been associated with prolonged progression-free survival and improved tolerability and health-related quality of life, as compared with platinum-based doublet chemotherapy (6,7).

Icotinib hydrochloride (BPI-2009H), an orally active, EGFR-TKI, has shown similar antitumor activity to gefitinib and erlotinib in patients with advanced NSCLC (8,9). Based on preclinical and clinical data, icotinib has been shown to inhibit the growth of human tumor cell lines that over express EGFR and has a high level of tolerance among healthy Chinese subjects (10).

As the toxicity of TKIs is less than that of cytotoxic agents, their utility as a first-line treatment for patients with NSCLC with poor PS has been studied. Patients of East-Asian origin with adenocarcinoma have been shown to be significantly associated with a favorable response to EGFR TKIs (4,5).

The present study proposed that icotinib would confer a survival advantage as a first-line therapy, compared with BSC, if eligible patients were selected on the basis of their histology. This retrospective study was conducted to evaluate the efficacy, toxicity and feasibility of first-line icotinib treatment for patients with adenocarcinoma of the lung together with extremely poor PS, who would not be eligible candidates for standard therapy.

## Materials and methods

### Patients

The medical charts of all patients with adenocarcinoma of the lung who received icotinib from May 1, 2011 to October 31, 2012 at the Zhejiang Cancer Hospital (Hangzhou, China), were reviewed. Of the 174 lung adenocarcinoma patients treated with icotinib, 42 cases were treated as first-line due to poor PS, with no indication for routine therapy such as surgical intervention, chemotherapy or radiotherapy. The patients were aged from 35 to 85 years, with a median age of 62.5 years. Each patient was evaluated, which included clinical history and physical examination, computed tomography (CT) of the chest, hematology and blood chemistry profiles prior to treatment. The study was approved by the ethics committee of Zhejiang Cancer Hospital.

### Pathological analysis

Lung adenocarcinoma was confirmed either histologically or cytologically. Cytological specimens were obtained from the sputum, bronchial biopsy, pleural effusion and needle aspiration biopsy. Mutations in the extracted DNA of eight specimens from 42 NSCLC patients were examined by polymerase chain reaction-based direct sequencing for EGFR (exons 19 and 21).

### Drug administration

Icotinib (125 mg) was orally administered three times per day (patent no. WO2003082830; Zhejiang Bata Pharma Ltd., Hangzhou, China). Tablets were taken 1 h before or after eating until disease progression or undue toxicity was observed. For patients with severe toxicity, the icotinib dosing schedule could be decreased to twice per day. Second-line chemotherapy or other treatments following the termination of icotinib therapy were permitted.

### Clinical assessment

The objective tumor responses were evaluated as the complete response (CR), partial response (PR), stable disease (SD) or progressive disease (PD), in accordance with the Response Evaluation Criteria in Solid Tumors (11). Disease control was defined as the complete response + partial response + stable disease, which was confirmed and sustained for 4 weeks or longer. Baseline assessments were performed within 28 days of commencement of the treatment. Assessments were performed every 4 weeks for the first 4 months and then every 8 weeks until disease progression. All adverse events were reported and graded according to the National Cancer Institute Common Toxicity Criteria (version 3.0) (12). Data were also collected when icotinib treatment was interrupted or withdrawn due to adverse events. Changes in the PS scores during the course of the treatment were compared with the baseline score. Routine clinical and laboratory assessments were performed at least every 4 weeks.

### Statistical analyses

The primary endpoint was to evaluate the progression-free survival (PFS). PFS was defined as the interval between the start of the treatment and the date of the first observation of disease progression or death from any cause. Secondary endpoints were the overall survival and response rate. Overall survival (OS) was assessed from the date of icotinib treatment until death from any cause. PFS and OS were estimated using the Kaplan-Meier method, and the differences between subgroups were analyzed by the log-rank statistic. The strata were smoking history, performance status, and gender. Tertiary endpoints were PS improvement rate and toxicity. P<0.05 was considered to indicate a statistically significant difference. SPSS, version 13.0 (SPSS, Inc., Chicago, IL, USA) was used for statistical analyses.

## Results

### Patient characteristics

A total of 510 patients with NSCLC were screened between May 1, 2011 and October 31, 2012. Among them, 174 lung adenocarcinoma patients were treated with icotinib, and 42 patients with poor PS entered this study (Table I). The majority of patients had stage IV disease, and 11/42 (26.2%) patients had multiple sites of distant metastases. Thirty-two patients had Eastern Cooperative Oncology Group (ECOG) PS 3 or 4 due to various cancer-related conditions, including respiratory failure owing to multiple pulmonary metastasis, carcinomatous lymphangiosis, malignant pleural effusion and oxygen dependence. A total of 32 patients, <75 years old, had ECOG PS 3 to 4; nine cases, 75 to 79 years-of-age, had ECOG PS 2; and one patient, 80-years-old, had ECOG PS 1. Heavy smokers (defined as >10 pack-years) were included in the study, although the majority of patients with adenocarcinomas were non- or light-smokers. EGFR mutation analysis was performed on eight patients before the treatment, of which two had an exon 19 deletion, and one had an L858R mutation.

### Response and survival

The objective tumor responses are summarized in Table II. The overall response (OR) and disease control rates were 33.3 and 85.7%, respectively. Stratified analyses were performed to examine the differences in response rate between certain clinical factors. The analyses revealed that gender, smoking status and PS had no association with icotinib response. For the two patients who had EGFR mutations, both achieved PR and longer PFS of more than 12 months, which were significantly more effective as compared with those with wild-type EGFR (where only 50% achieved SD and the median PFS was seven months).

**Figure 1:**
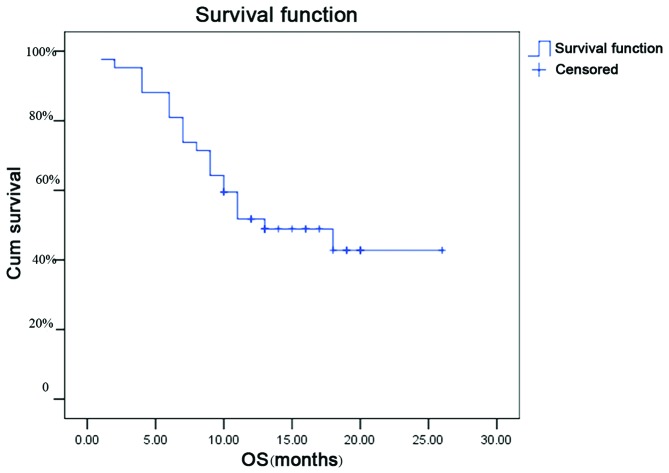
Kaplan-Meier estimates of OS for all patients from start of treatment. The median OS time was 13.0 months. Crosses indicate censored data. OS, overall survival; Cum survival, cumulative survival.

**Figure 2:**
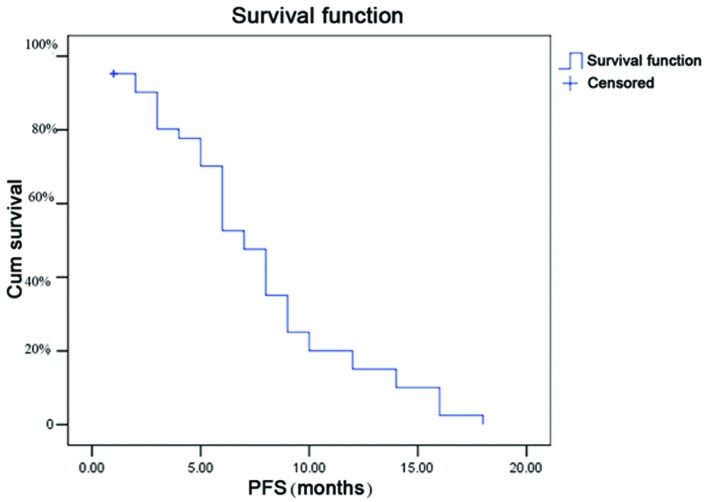
Kaplan-Meier estimates of PFS. The median PFS time was 7.0 months. Crosses indicate censored data. PFS, progression-free survival; Cum survival, cumulative survival.

The follow-up time was 12 to 26 months (median, 17.8 months), and 20/42 patients were alive on the last day of the follow up. The median survival time was 13.0 months (95% CI, 5.6–20.4; Fig. 1), the median PFS time was 7.0 months (Fig. 2) and the 1-year survival rate was 71.4%.

### Improvement of PS

Thirty-three (79%) of 42 patients had a significant improvement in their PS following icotinib treatment (Wilcoxon signed rank test, P=0.005).

### Toxicity

The toxicities that occurred during the icotinib treatment are listed in Table III. The majority of the adverse events were grade 1 or 2. The most frequent adverse events were rash, diarrhea and fatigue. Two patients had grade 2 or worse AST/ALT elevation and one patient died of grade 4 interstitial lung disease. No hematological toxicity was observed in any of the patients. Reduction of the dosage or discontinuation of icotinib was not required in any of the patients.

## Discussion

The lack of effective therapies in patients with advanced NSCLC and extremely poor PS (particularly PS 3–4) is a major clinical problem. EGFR gene family members have been shown to be widely expressed in various human cancers, including breast, head and neck, NSCLC and ovarian cancers (1). Gefitinib is a highly specific EGFR kinase inhibitor. However, no clinical benefit was identified in a randomized trial comparing gefitinib with BSC for unselected patients with NSCLC exhibiting poor PS (13). By selecting patients with adenocarcinoma who had never smoked, the administration of gefitinib in patients with advanced or metastatic NSCLC had a marked OR of ~55.6% (14), which was comparable to gefitinib for EGFR mutation-positive patients with PS 0–2 (15–20).

Icotinib is a small-molecule inhibitor of EGFR tyrosine kinase, with a similar chemical structure and active mechanism to gefitinib (9), and was recently approved by the State Food and Drug Administration of China (http://app1.sfda.gov.cn/datasearch/face3/base.jsptatement.). The current retrospective study demonstrated that oral administration of icotinib was well tolerated in patients with lung adenocarcinoma with a poor PS. The median PFS time (7.0 months) was markedly improved as compared with that expected in patients managed only with BSC (7). The PFS time reported in the present study was improved over that reported previously for unselected patients undergoing standard chemotherapy with PS 2 treated with erlotinib (median PFS time, 1.9 to 2.9 months). Only a few male patients were enrolled in this study; therefore, precaution should be taken in interpreting this data, since female patients have been shown to survive longer than male patients (21,22).

In the present study, two patients with EGFR exon 19 or 21 mutations had a PR. This data is inconsistent with the majority of previous studies (6,7); however, the sample numbers were low due to poor PS or older age of the patients, though the assessment of EGFR mutation status before treatment is considered to be reasonable and predictive.

In the present study, the most frequent toxicities were rashes and diarrhea, both of which were grade 1 and 2. One patient, however, succummbed to uncontrollable severe interstitial pneumonia.

In conclusion, icotinib is active in Chinese patients with lung adenocarcinoma with poor PS. Data from the present study suggests that icotinib should not be used in patients with interstitial lung disease or concurrent with uncontrolled pneumonia. Further randomized trials are required to delineate the role of icotinib as a first-line treatment in this subset of selected patients as compared with BSC or other TKIs.

## Figures and Tables

**Table I:** Patient characteristics.

Characteristic	n (%)
Age, years^a^	62.5 (35–85)
Gender	
Male	7 (16.7)
Female	35 (83.3)
ECOG performance status	
0–1	1 (2.4)
2	9 (21.4)
3–4	32 (76.2)
Smoking status, pack-years	
0	34 (84.0)
1–19	3 (7.1)
≥20	5 (11.9)
Stage	
I	1 (2.4)
II	1 (2.4)
III	5 (11.9)
IV	35 (83.3)
Metastatic site	
Lung	13 (31.0)
Bone	14 (33.3)
Liver	8 (19.0)
Brain	5 (11.9)
Malignant pleural effusion	7 (16.7)
Other	4 (9.5)
EGFR	
Mutation	2 (4.8)
Wild type	6 (14.3)
Unknown	34 (81.0)

a Median (range).

ECOG, Eastern Cooperative Oncology Group; EGFR, epidermal growth factor receptor.

**Table II:** Patient responses to treatment.

Response	No. of patients	Response rate, %
Complete response	0	0
Partial response	14	33.3
Stable disease	22	52.4
Progressive disease	6	13.3
Overall response	14	33.3
Disease control rate	36	85.7

**Table III:** Toxicities related to the treatment.

Toxicity	Grade				Total no., %

	1	2	3	4	
Rash	8	5	2	0	35.7
Dry skin	9	6	1	0	38.1
Mucositis	1	3	0	0	9.5
Anorexia	2	2	0	0	9.5
Fatigue	5	1	0	0	14.3
Diarrhea	8	7	3	1	45.2
Vomiting	2	1	0	0	7.1
Lung	0	0	0	1	2.3
Increased ALT	2	1	0	0	7.1

ALT, alanine transaminase.
